# MG53 Protects against Sepsis-Induced Myocardial Dysfunction by Upregulating Peroxisome Proliferator-Activated Receptor-*α*

**DOI:** 10.1155/2020/7413693

**Published:** 2020-08-27

**Authors:** Xue Han, Daili Chen, Ning Liufu, Fengtao Ji, Qingshi Zeng, Weifeng Yao, Minghui Cao

**Affiliations:** ^1^Department of Anesthesiology, Sun Yat-sen Memorial Hospital, Sun Yat-sen University, Guangzhou 510120, China; ^2^Department of Anesthesiology, Affiliated Shenzhen Maternity & Child Healthcare Hospital, Southern Medical University, Shenzhen, 518028 Guangdong, China; ^3^Department of Anesthesiology, Cardiovascular Institute of Guangdong Province, Guangdong Provincial People's Hospital, Guangzhou, Guangdong 510030, China; ^4^Department of Anesthesiology, The Third Affiliated Hospital of Sun Yat-sen University, Guangzhou 510630, China

## Abstract

**Background:**

The heart is one of the most commonly affected organs during sepsis. Mitsugumin-53 (MG53) has attracted attention in research due to its cardioprotective function. However, the role of MG53 in sepsis-induced myocardial dysfunction (SIMD) remains unknown. The purpose of this study was to explore the underlying mechanism of MG53 in SIMD and investigate its potential relationship with peroxisome proliferator-activated receptor-*α* (PPAR*α*).

**Methods:**

The cecal ligation and puncture (CLP) model was created to induce SIMD in rats. Protein levels of MG53 and PPAR*α*, cardiac function, cardiomyocyte injury, myocardial oxidative stress and inflammatory indicators, and cardiomyocyte apoptosis were measured at 18 h after CLP. The effects of MG53 on PPAR*α* in SIMD were investigated via preconditioning recombinant human MG53 (rhMG53) and PPAR*α* antagonist GW6471.

**Results:**

The expression of MG53 and PPAR*α* sharply decreased in the myocardium at 18 h after CLP. Compared with the sham group, cardiac function was significantly depressed, which was associated with the destructed myocardium, upregulated oxidative stress indicators and proinflammatory cytokines, and excessive cardiomyocyte apoptosis in the CLP group. Supplementation with rhMG53 enhanced myocardial MG53, increased the survival rate with improved cardiac function, and reduced oxidative stress, inflammation, and myocardial apoptosis, which were associated with PPAR*α* upregulation. Pretreatment with GW6471 abolished the abovementioned protective effects induced by MG53.

**Conclusions:**

Both MG53 and PPAR*α* were downregulated after sepsis shock. MG53 supplement protects the heart against SIMD by upregulating PPAR*α* expression. Our results provide a new treatment strategy for SIMD.

## 1. Introduction

Sepsis is a life-threatening organ dysfunction caused by the uncontrolled response of a host's anti-infective immunity [1]. According to the US Centers for Disease Control's (CDC) estimation in 2019, sepsis contributes to the death of approximately 270,000 Americans annually [2], and this figure is at least 6 million per year globally [1]. Evans [3] simplified this complex pathophysiological process into four main features: endothelial dysfunction, coagulopathy, cellular dysfunction, and cardiovascular dysfunction. This demonstrates that the cardiovascular system acts as a pivotal area in the pathogenesis of sepsis, and the heart is the most vulnerable organ. Approximately 40~60% of patients with sepsis exhibit signs of myocardial dysfunction, and mortality rates are remarkably increased in these patients compared to those without myocardial dysfunction (70% *vs.* 20%) [4]. Sepsis-induced myocardial dysfunction (SIMD) is defined as the intrinsic myocardial systolic and diastolic dysfunction of both the left and right sides of the heart, induced by sepsis [5].

It is generally accepted that SIMD is a result of the interaction of uncontrolled inflammation, autonomic malfunction, intracellular Ca^2+^ transporter dysfunction, energetic starvation, mitochondrial dysfunction, oxidant/antioxidant imbalance, and excessive cardiomyocyte apoptosis [6]. The pathophysiologic process of SIMD is complicated; thus, there is no expert consensus guideline for its diagnosis and treatment at present [4]. Although the Surviving Sepsis Campaign (SSC) guidelines [7, 8] propose a therapeutic schedule for sepsis and its complications, mortality remains high [9]. Currently, SIMD therapies are restricted to symptomatic support, such as anti-infection, fluid resuscitation, and oxygen delivery amelioration [7, 8]. Attempts to develop molecular-targeted therapy have failed clinically [10]. Therefore, the personalized treatment of SIMD is still lacking.

Mitsugumin-53 (MG53) is a member of the muscle-specific tripartite motif (TRIM) family that plays an essential role in plasma membrane damage repair [11]. It is rapidly recruited when cell membranes are attacked, following which it facilitates the movement of membrane-repair vesicles toward the damage site and binds exposed phosphatidylserine at the damaged lipid bilayer [12]. Pathogenic factors, such as Ca^2+^ influx and oxidant entry into the cytoplasm, are prevented when membrane disruption is ended. Both in vivo and in vitro, MG53 has demonstrated a protective effect against membrane disruption associated with muscular dystrophy [13], acute kidney [14] and lung [15] injury, ischemic [16] and traumatic [17] brain injury, and myocardial infarction (MI) [18]. We found that MG53 is anchored by dysferlin, which reduces oxidative stress and hepatocyte apoptosis in a hepatic ischemic/reperfusion injury (HIRI) model [19]. MG53 interacts with p85 and caveolin 3 (CaV3) and activates the prosurvival reperfusion injury salvage kinase (RISK) pathway (PI3K/Akt/GSK-3*β* cascade and ERK_1/2_ pathway) in cardiac ischemic preconditioning (IPC) or postconditioning (IPO) [20, 21]. Genetic deletion of MG53 results in damage to cell membrane integrity and impaired myocardial regeneration [20–22].

However, Liu et al. [23], using transgenic mice, showed that the cardiac-specific upregulation of MG53 induced diabetic cardiomyopathy (DCM) via transcriptional activation of peroxisome proliferator-activated receptor-*α* (PPAR*α*). Interestingly, PPAR*α* has been proven to support heart function during SIMD [24]. However, the relationship between MG53 and PPAR*α* in SIMD is an enigma. Therefore, we designed the current study to investigate the role of MG53 on SIMD and the underlying mechanisms of action.

## 2. Materials and Methods

### 2.1. Animals and Operative Procedure

This study was approved by the Animal Care Committee, Sun Yat-Sen University, China. In total, 172 adult Sprague-Dawley rats (240~270 g) were purchased from the Laboratory Animal Center of Sun Yat-Sen University and housed in individual cages in the specific pathogen-free (SPF) experimental room for acclimatization one week before the study. Rats were given ad libitum access to a standard diet and water under a controlled temperature (21~23°C), humidity (20~30%), and alternating light cycle (12 h light/dark) environment. Food was removed 8 h before the study, but free access to water remained. All the experimental procedures were strictly performed in accordance with the *Guide for the Care and Use of Laboratory Animals* (1996). All possible efforts were made to minimize the number of animals used and their suffering. Cecal ligation and puncture (CLP) operation was performed as described in previous studies [25]. Rats were positioned on a homoeothermic heating pad to maintain a rectal temperature at 37 ± 1°C and inhaled a gas mixture containing 3% isoflurane and 40% oxygen for general anesthesia during the surgery. A midline laparotomy (approximately 3 cm long incision) was performed on the anterior abdomen after disinfection with 10% povidone iodine. The cecum was exposed and ligated by a 3-0 silk suture just below the ileocecal valve to maintain bowel continuity. The cecum was punctured twice with an 18-gauge needle, and a small amount of bowel content was expelled from the punctures by manual application of sufficient pressure. Then, the cecum was returned to the peritoneal cavity, and the abdominal incision was closed with 4-0 silk suture in two layers. At the end of surgery, each rat received a subcutaneous injection of physiological saline solution (5 ml/kg), then returned to ambient air. For the sham-operated animals, the cecum was mobilized but neither ligated nor punctured.

### 2.2. Experimental Protocol and Drug Administration

**Figure 1:**
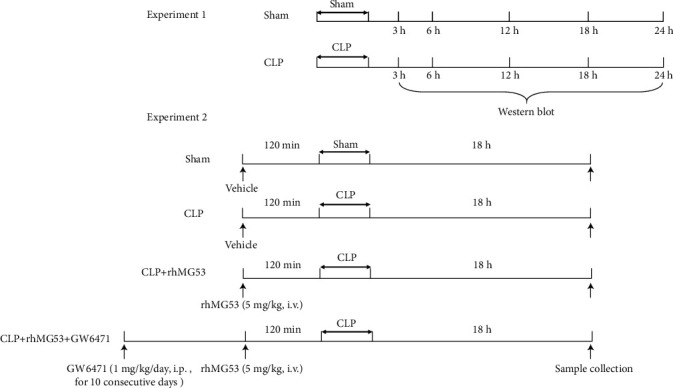
Protocol for experiments. CLP = cecal ligation and puncture; rhMG53 = recombinant human MG53.

As the schematic diagram is shown in Figure 1, two experiments were performed. In experiment 1, a total of 80 rats were randomly assigned to receive sham or CLP operation. At 3 h, 6 h, 12 h, 18 h, and 24 h after the operation, the myocardial tissues from the left ventricle (LV) were collected to measure the expression trend of MG53 and PPAR*α* by western blotting (*n* = 8).

In experiment 2, a total of 32 rats were randomly allocated to four groups (*n* = 8), consisting of a sham group, CLP group, CLP+recombinant human MG53 (rhMG53) group, and CLP+rhMG53+GW6471 group. Animals received a sham operation in the sham group and CLP operation in the other three groups. rhMG53 protein was administered (5 mg/kg, i.v.) in the CLP+rhMG53 and CLP+rhMG53+GW6471 groups 120 min before CLP surgery [16] while the sham and CLP groups were administered isopycnic vehicle (0.9% sterile saline). Rats in the CLP+rhMG53+GW6471 group also received 1 mg/kg/day i.p. GW6471 (Sigma, St. Louis, MO, USA), a PPAR*α* antagonist, for 10 consecutive days before surgery [26]. The preparation, separation, and purification of rhMG53 protein have been described in our previous study [19].

### 2.3. Cardiac Function Evaluation

Then, 18 h after the operation, a hypodynamic stage of sepsis [27], the cardiac function of rats was assayed. After anesthesia by 3% isoflurane inhalation, the LVs were intubated via the right common carotid artery by a catheter filled with heparin saline (500 U/ml) to measure mean arterial blood pressure (MABP) and LV pressure. Left ventricular systolic pressure (LVSP), left ventricular end-diastolic pressure (LVEDP), and maximum rise/fall rate of LV pressure (±dp/dt_max_) were recorded by the PowerLab Data Acquisition and Analysis System (ADInstruments, Australia).

### 2.4. Sample Collection

In experiment 1, the LVs were snap-frozen in liquid nitrogen, then stored at -80°C for western blotting at 3 h, 6 h, 12 h, 18 h, and 24 h after the CLP.

In experiment 2, immediately after cardiac function evaluation, the common carotid artery was punctured by a disposable needle for blood collection (5 ml). Blood samples were layered at room temperature (26°C) and centrifuged at 4°C 3000 × rpm for 10 min to collect the serum for subsequent detections. Then, the rats were euthanized by excessive anesthesia administration. Myocardial tissues from the lower third apex of the LV were selected. One-third of these tissues was postfixed by 10% paraformaldehyde at 4°C for 48 h, paraffin-embedded, and sectioned to a thickness of 5 *μ*m. Another third was collected for H-FABP and oxidative stress biomarker detection. The final third was snap-frozen in liquid nitrogen, then stored at -80°C for western blotting.

### 2.5. Hematoxylin and Eosin (H&E) Staining

Sections were stained using a H&E Staining Kit (Abcam, Cambridge, MA, USA), according to the manufacturer's instructions. Morphological changes were evaluated by light microscopy (Nikon Corporation, Japan) at 200x magnification.

### 2.6. Myocardial Injury Marker Detection in Serum

An enzyme-linked immunosorbent assay (ELISA) was performed to measure the levels of cardiac troponin I (cTnI) (Abcam, Cambridge, UK) and creatine kinase isoenzyme MB (CK-MB) (MyBioSource, Inc., San Diego, CA, USA) in serum. The lactate dehydrogenase (LDH) activity in serum was determined by chemical colorimetry using an LDH Cytotoxicity Assay Kit (Cayman Chemical Company, Ann Arbor, Michigan, USA).

### 2.7. Myocardial Heart-Type Fatty Acid Binding Protein (H-FABP) Detection

Myocardial tissues (approximately 0.2 g) were cut with ophthalmic scissors, dissolved in 4x RIPA lysate, and homogenized using an electric homogenizer to obtain the supernatant. Quantitative analysis of H-FABP levels was performed using an ELISA kit (MyBioSource, Inc., San Diego, CA, USA) with a sensitivity of 45~1200 pg/ml.

### 2.8. Detection of Superoxide Production in Heart Tissues

Fresh LVs were embedded in Tissue-Tek® O.C.T. Compound (VWR, Atlanta, GA, USA) and sectioned (5 *μ*m) onto slides for fluorescent staining with dihydroethidium (DHE, 10 *μ*M; Beyotime, China). Images of LVs were immediately acquired and assessed at 400x using Image-Pro Plus software (Version 6.0, Media Cybernetics, USA) linked to a Ti-S inverted fluorescence microscope (Nikon, Japan). DHE fluorescent intensity was quantified as folds of the sham group.

### 2.9. Assay of Oxidative Stress Biomarkers in Myocardium

LVs were homogenized and centrifuged to determine the concentrations of the antioxidative and oxidative indicators. The level of superoxide dismutase (SOD) was determined via a colorimetric method using a SOD assay kit with WST-1 (BioVision, Inc., Milpitas, California, USA). The concentration of glutathione (GSH) was detected via the DTNB colorimetric method using the detection kit (Geno Technology, Inc., MO, USA). The level of malondialdehyde (MDA) was detected via the thiobarbituric acid colorimetric method using a Lipid Peroxidation (MDA) Assay Kit (Abcam, Cambridge, MA, USA). The activity of free 15-F2t-isoprostane was measured using a 15-Isoprostane F2t ELISA Kit (Neogen Co., Lexington, KY, USA). All procedures were conducted in strict accordance with the manufacturer's instructions. All samples were measured in triplicate, and the results were averaged.

### 2.10. Masson's Trichrome Staining

Slides were stained with Masson's trichrome stain (Masson) kit (Sigma-Aldrich, Saint Louis, MO, USA), as per the manufacturer's instructions. Figures were captured at 400x magnification with a light microscope (Nikon, Tokyo, Japan) and analyzed with Image-Pro Plus software (Version 6.0, Media Cybernetics, USA) for semiquantitative analysis by two persons blinded to the experimental design. Five different fields for each section were randomly selected for analysis, and the average value was taken as the final value for the samples.

### 2.11. Cytokine Measurement by ELISA

Inflammatory cytokines, interleukin-1*β* (IL-*β*), IL-6, and tumor necrosis factor-*α* (TNF-*α*) in serum were measured using ELISA kits, according to the manufacturer's instructions (R&D Systems Inc., Minneapolis, USA). The concentrations of the cytokines were quantified by referring to standard curves.

### 2.12. TdT-Mediated dUTP Nick-End Labeling (TUNEL) for DNA Fragmentation

Extensive DNA degradation is the signature of the late stage of apoptosis. TUNEL staining (horseradish peroxidase method) was conducted to detect apoptotic myocardial cells. All procedures were conducted in strict accordance with the manufacturer's instructions of the TACS® TdT In Situ Apoptosis Detection Kit-DAB (R&D Systems Inc., Minneapolis, USA). Figures were immediately acquired at 400x magnification by a light microscope (Nikon, Tokyo, Japan). The proportion of TUNEL-positive cells was counted using Image-Pro Plus software (Version 6.0, Media Cybernetics, USA) by two persons blinded to the experimental design. Five different fields of each section were randomly selected for analysis, and the average value was taken as the final value of the sample.

### 2.13. Western Blot Analysis

Myocardial tissues were homogenized, and protein concentrations were determined using the BCA protein assay (Bio-Rad, Hemel Hempstead, Herts, UK). In total, 60 *μ*g of each sample was subjected to western blotting using the following primary antibodies incubated overnight at 4°C: anti-MG53 at 1 : 1000 dilution, anti-PPAR*α* at 1 : 2000 dilution, anti-cyclooxygenase-2 (COX-2) at 1 : 2000 dilution, anti-caspase-3 (proform and the p17 cleaved form) (#9662, Cell Signaling Technology, Danvers, MA, USA) at 1 : 1500 dilution, anti-Bax at 1 : 1000 dilution, anti-Bcl-2 at 1 : 1000 dilution, and anti-*β*-actin at 1 : 2000 dilution. The secondary antibodies were goat-anti-rabbit IgG at 1 : 5000 dilution. All antibodies except anti-caspase-3 were purchased from Abcam Inc., Cambridge, MA, USA. Immunoreactive proteins were visualized using the ECL Western Blot System (Pierce Biotechnology, Rockford, IL, USA) and detected by a Tanon 5500 imaging system (Tanon, Shanghai). Images were analyzed using ImageQuant TL software v2003.03 (GE Healthcare, Milwaukee, WI, USA). The band density of the target proteins was normalized to those of the corresponding *β*-actin and expressed as fractions of the control sample from the same gels.

### 2.14. Survival Analysis

Rats (*n* = 15) under the same treatments in experiment 2 (Figure 1) were used to evaluate the survival rate. Animals in each group were kept under SPF conditions and monitored via video. All rats had free access to food and water. From the onset of operations, animals were monitored via a video camera. The survivors were transferred to their individual cages and had free access to food and water. The survival rate was evaluated within 72 h.

### 2.15. Statistical Analysis

The data were expressed as mean ± standard deviation (SD) and analyzed by one-way ANOVA after a homogeneity test for variance, followed by Tukey's test. Statistical analyses were conducted using GraphPad Prism 8.0 software, and *P* < 0.05 in two-tailed testing was considered statistically significant.

## 3. Results

### 3.1. Myocardial MG53 and PPAR*α* Decreased in SIMD Rats: Supplementation of MG53 Improved PPAR*α* Expression

MG53 is abundantly expressed in rodent cardiac muscle tissues under normal circumstances [28]. However, the expression of MG53 and PPAR*α* in SIMD is unknown.

**Figure 2:**
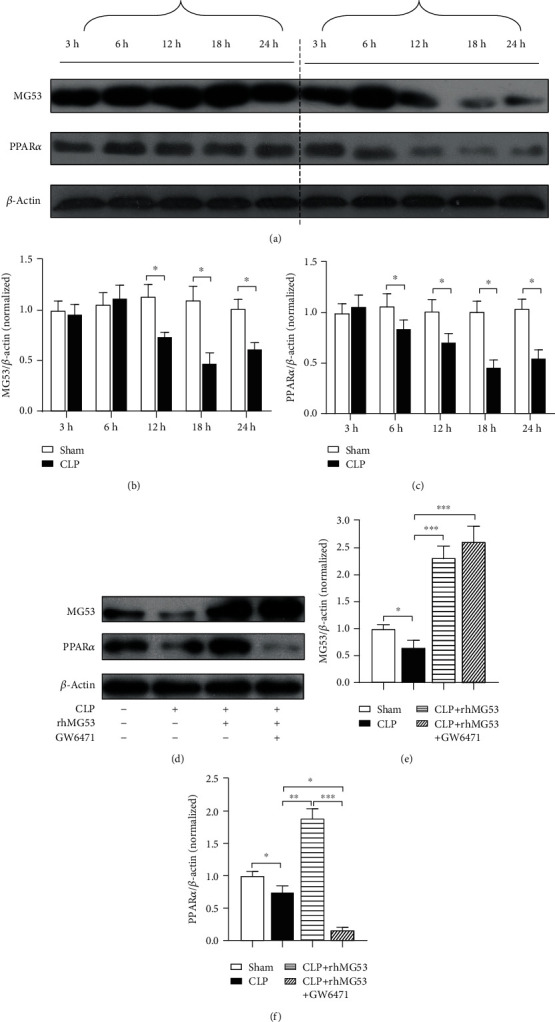
Myocardial MG53 and PPAR*α* decreased in SIMD rats. Supplementation of MG53 improved PPAR*α* expression. (a) Representative western blot bands of MG53 and PPAR*α* at 3 h, 6 h, 12 h, 18 h, and 24 h after CLP in experiment 1. Densitometric analysis of the (b) MG53 and (c) PPAR*α* protein levels in experiment 1. (d) Representative western blot bands of MG53 and PPAR*α* at 18 h after CLP in experiment 2. Densitometric analysis of the (e) MG53 and (f) PPAR*α* protein levels in experiment 2. Data are presented as mean ± SD, *n* = 8. ^∗^*P* < 0.05, ^∗∗^*P* < 0.01, and ^∗∗∗^*P* < 0.001. CLP = cecal ligation and puncture; rhMG53 = recombinant human MG53.

As shown in Figures 2(a)–2(c), we identified a decreasing trend of MG53 and PPAR*α* in rat myocardial tissue after CLP surgery via western blotting. The protein levels of MG53 and PPAR*α* reached to the lowest point at 18 h after CLP (both *P* < 0.05*vs.* sham group) and improved slightly at 24 h after CLP (both *P* < 0.05*vs.* sham group). So, we chose the point-in-time, 18 h after CLP, to investigate the relationship between MG53 and PPAR*α* in SIMD. Then, we supplemented myocardial MG53 via intravenous injection of rhMG53 and selected the GW6471 precondition group as a control. MG53 administration significantly increased myocardial PPAR*α* protein levels (*P* < 0.01*vs.* CLP group) in SIMD rats. These results suggest that MG53 may be effective in enhancing PPAR*α* expression in the myocardium of septic rats.

### 3.2. MG53 Improved the Cardiac Function and Survival Rate in SIMD Rats, Which Was Exacerbated by GW6471

**Figure 3:**
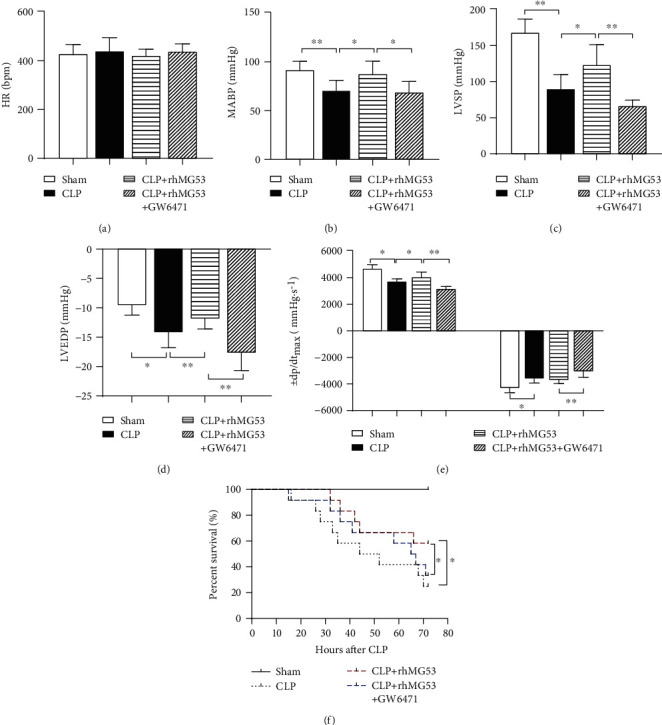
MG53 improved cardiac function and survival rate in SIMD rats, which was exacerbated by GW6471. Cardiac function indexes were demonstrated by (a) heart rate (HR), (b) mean arterial blood pressure (MABP), (c) left ventricular systolic pressure (LVSP), (d) left ventricular end-diastolic pressure (LVEDP), and (e) maximum rate of rise/fall of left ventricle pressure (±dp/dt_max_) in four groups. (f) Survival analysis. Data are presented as mean ± SD, *n* = 8 in cardiac function evaluation, *n* = 15 in survival rate evaluation. ^∗^*P* < 0.05, ^∗∗^*P* < 0.01, and ^∗∗∗^*P* < 0.001. CLP = cecal ligation and puncture; rhMG53 = recombinant human mitsugumin-53.

SIMD is characterized by cardiovascular disturbances and high mortality rates [29]. As shown in Figures 3(b)–3(e), rat cardiac functions were impaired after septic shock. In the CLP group, MABP (*P* < 0.01*vs.* sham group), LVSP (*P* < 0.01*vs.* sham group), and +dp/dt_max_ (*P* < 0.05*vs.* sham group) significantly decreased while LVEDP (*P* < 0.05*vs.* sham group) and −dp/ds_max_ (*P* < 0.05*vs.* sham group) significantly increased, indicating a decline in myocardial systolic and diastolic functions. MG53 relieved the cardiac function damage demonstrated by the amelioration of MABP, LVSP, and +dp/dt_max_ (all *P* < 0.05*vs.* CLP group) and the reduction of LVEDP (*P* < 0.01*vs.* CLP group). On the contrary, GW6471 abolished the protective effects of rhMG53, which manifested as a deterioration of MABP (*P* < 0.05*vs.* CLP+rhMG53 group), LVSP (*P* < 0.01*vs.* CLP+rhMG53 group), and +dp/dt_max_ (*P* < 0.01*vs.* CLP+rhMG53 group) and increased LVEDP and −dp/ds_max_ (both *P* < 0.01*vs.* CLP+rhMG53 group). There was no significant difference in HR among the four groups (Figure 3(a)).

As shown in Figure 3(f), the survival rate dropped by approximately 8.3% in both the CLP and CLP+rhMG53+GW6471 groups in 24 h after septic shock and continued to decline sharply. The mortality rates at 72 h were 0%, 75.0%, 41.7%, and 66.7% in the sham, CLP, CLP+rhMG53, and CLP+rhMG53+GW6471 groups, respectively. MG53 administration prolonged the survival time, and the first drop in survival rate was observed at 32 h after CLP, and 41.7% of rats survived at 72 h (*P* < 0.05*vs.* CLP group). This protective effect was reversed by GW6471, wherein the first drop in survival rate was observed at 15 h after CLP and only 33.3% of rats survived at 72 h (*P* < 0.05*vs.* CLP+rhMG53 group).

### 3.3. MG53 Prevented Cardiomyocyte Injury in SIMD Rats, Which Was Reversed by GW6471

**Figure 4:**
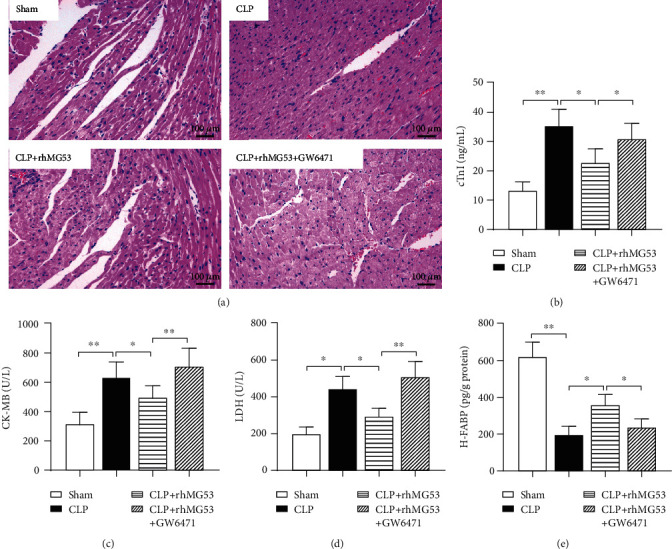
MG53 prevented cardiomyocyte injury in SIMD rats, which was reversed by GW6471. Myocardium architecture was visualized by (a) H&E staining (200x magnification, scale bar = 100 *μ*m). Myocardial injury was detected through serum levels of (b) cardiac troponin I (cTnI), (c) creatine kinase isoenzyme MB (CK-MB), (d) lactate dehydrogenase (LDH), and (e) heart-type fatty acid-binding protein (H-FABP) by the ELISA method. Data are presented as mean ± SD, *n* = 8. ^∗^*P* < 0.05, ^∗∗^*P* < 0.01, and ^∗∗∗^*P* < 0.001. CLP = cecal ligation and puncture; rhMG53 = recombinant human mitsugumin-53.

The integrity of cardiomyocytes was destroyed, and the myocardial injury marker levels were elevated in serum when SIMD occurred [4]. As shown in Figure 4(a), the clear myocardial striation and neatly arranged cells demonstrate the normal architecture of the myocardium in the sham group. On the contrary, the cardiomyocytes were degenerated, myocardial fibers were disorganized, and interstitium was edematous, congested, and infiltrated with inflammatory cells in the CLP group. MG53 prevented morphological deformation in SIMD rats, which was reversed by GW6471. As shown in Figures 4(b)–4(d), serum myocardial injury and necrosis markers, cTnI (*P* < 0.01*vs.* sham group), CK-MB (*P* < 0.01*vs.* sham group), and LDH (*P* < 0.05*vs.* sham group), significantly increased 18 h after CLP. The MG53 supplement prevented the changes (all *P* < 0.05*vs.* CLP group), which were reversed by GW6471 (*P* < 0.05 in cTnI and *P* < 0.01 in CK-MB and LDH *vs.* CLP+rhMG53 group). H-FABP is a striated muscle abundant cytoplasmic protein that is rapidly released from cytoplasm at the very early stages of myocardial injury [30]. As shown in Figure 4(e), myocardial H-FABP was downregulated after CLP (*P* < 0.01*vs.* sham group). MG53 improved the levels of H-FABP in the myocardium (*P* < 0.05*vs.* CLP group), which were reversed by GW6471 (*P* < 0.05*vs.* CLP+rhMG53 group).

### 3.4. MG53 Attenuated Myocardial Oxidative Stress in SIMD Rats, Which Was Abolished by GW6471

**Figure 5:**
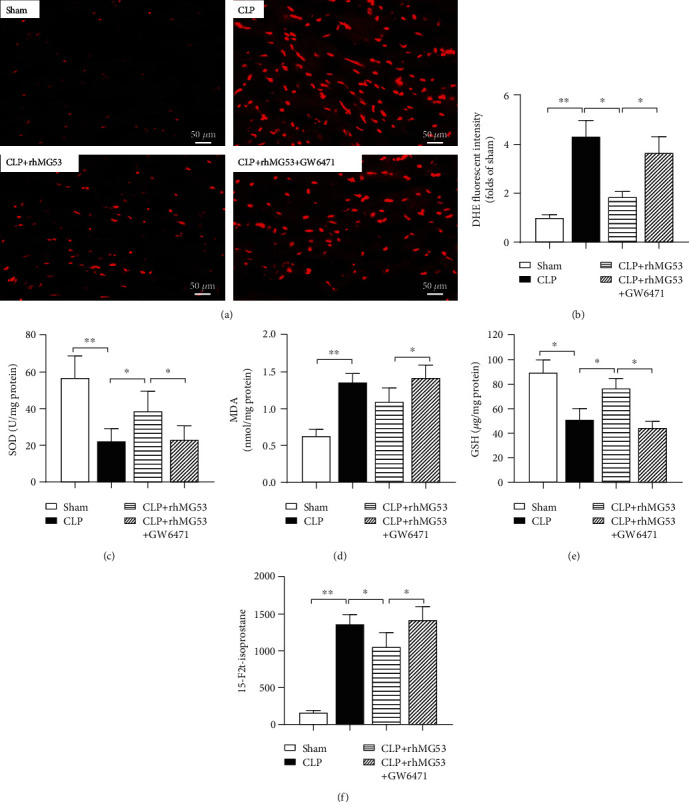
MG53 attenuated myocardial oxidative stress in SIMD rats, which was abolished by GW6471. Oxidative stress injury was detected through (a) representative DHE stained images depicting in situ superoxide production (red, 400x magnification, scale bar = 50 *μ*m), (b) quantification of DHE-signal intensity (folds of the sham group), (c) superoxide dismutase (SOD), (d) malondialdehyde (MDA), (e) glutathione (GSH) activity, and (f) free 15-F2t-isoprostane levels in the myocardium via an ELISA. Data are presented as mean ± SD, *n* = 8. ^∗^*P* < 0.05, ^∗∗^*P* < 0.01, and ^∗∗∗^*P* < 0.001. CLP = cecal ligation and puncture; rhMG53 = recombinant human mitsugumin-53.

As shown in Figures 5(a)–5(f), myocardial antioxidative indicators, SOD activity (*P* < 0.01*vs.* sham group), and GSH levels (*P* < 0.05*vs.* sham group) significantly decreased accompanied by increased oxidative indicators, DHE fluorescent intensity (*P* < 0.01*vs.* sham group), MDA (*P* < 0.01*vs.* sham group), and 15-F2t-isoprostane (*P* < 0.01*vs.* sham group), 18 h after CLP. These changes were attenuated by MG53 administration (*P* < 0.05 in SOD, GSH, DHE fluorescent intensity, and 15-F2t-isoprostane *vs.* CLP group) and aggravated by GW6471 (all *P* < 0.05*vs.* CLP+rhMG53 group).

### 3.5. MG53 Alleviated Myocardial Inflammation in SIMD Rats, Which Was Exacerbated by GW6471

**Figure 6:**
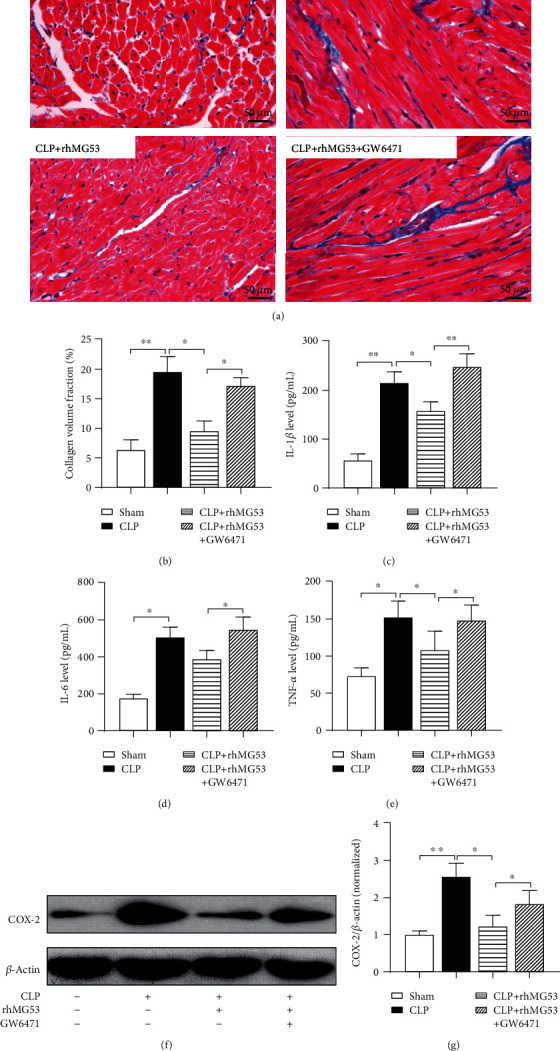
MG53 alleviated myocardial inflammation in SIMD rats, which was exacerbated by GW6471. Collagen accumulation was demonstrated by (a) Masson's trichrome staining. Collagen was stained blue in paraffin-embedded heart tissue sections (400x magnification, scale bar = 50 *μ*m) and (b) the quantitation of interstitial fibrosis area (%). Inflammatory cytokines (c) interleukin-1*β* (IL-*β*), (d) IL-6, and (e) tumor necrosis factor-*α* (TNF-*α*) in serum were detected by ELISA, and (f) cyclooxygenase-2 (COX-2) expression in the myocardium was detected by western blotting. Data are presented as mean ± SD, *n* = 8. ^∗^*P* < 0.05, ^∗∗^*P* < 0.01, and ^∗∗∗^*P* < 0.001. CLP = cecal ligation and puncture; rhMG53 = recombinant human mitsugumin-53.

SIMD presents inflammatory cardiomyopathy manifesting as subendocardial collagen deposition, myocardial hypertrophy, and fibrosis [31]. Several cytokines, such as IL-1, IL-6, and TNF-*α*, have been considered myocardium-depressing factors [4]. As shown in Figures 6(a) and 6(b), collagen volume significantly increased 18 h after CLP (*P* < 0.01*vs*. sham group) and was reduced by MG53 (*P* < 0.05*vs.* CLP group). GW6471 reversed the effect of MG53 demonstrated by profoundly increased cardiac collagen volume (*P* < 0.05*vs.* CLP+rhMG53 group). As shown in Figures 6(c)–6(g), serum proinflammatory cytokines IL-1*β* (*P* < 0.01*vs.* sham group), IL-6 (*P* < 0.05*vs.* sham group), and TNF-*α* (*P* < 0.05*vs.* sham group) and myocardial COX-2 protein levels (*P* < 0.01*vs.* sham group) significantly increased 18 h after CLP. This change in inflammatory biomarkers was attenuated by MG53 (*P* < 0.05 in IL-1*β*, TNF-*α*, and COX-2 *vs.* CLP group) and aggravated by GW6471 (*P* < 0.01 in IL-1*β*, *P* < 0.05 in IL-6, TNF-*α*, and COX-2 *vs.* CLP+rhMG53 group).

### 3.6. MG53 Prevented Cardiomyocyte Apoptosis in SIMD Rats, Which Was Abolished by GW6471

**Figure 7:**
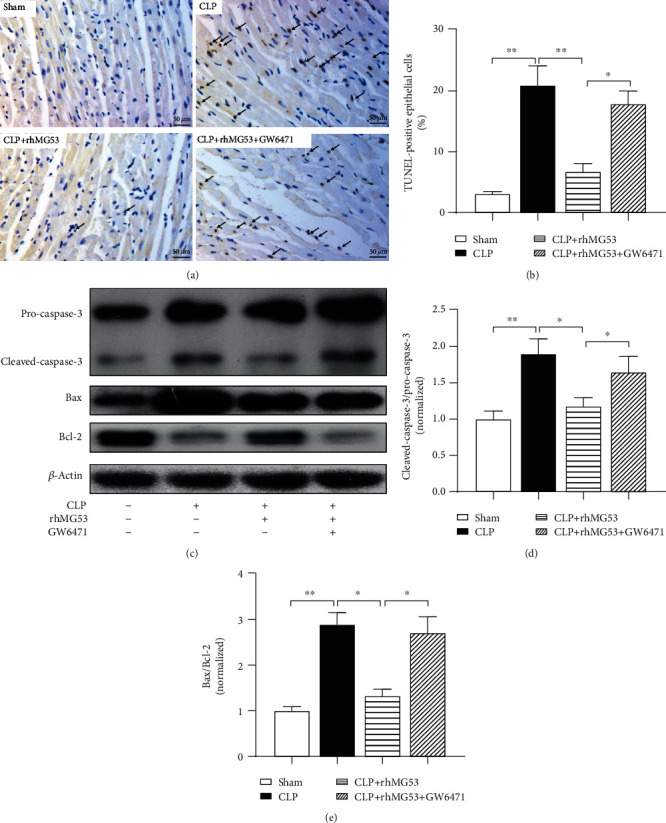
MG53 prevents cardiomyocyte apoptosis in SIMD rats, which was abolished by GW6471. Cardiomyocyte apoptosis was assessed by (a) terminal deoxynucleotidyl transferase dUTP nick-end labeling (TUNEL) (400x magnification, scale bar = 50 *μ*m) and (b) the quantitation of TUNEL-positive cells. TUNEL-positive cells were stained brown and are indicated by black arrows. (c) Representative western blot bands of pro-caspase-3, cleaved-caspase-3 (p17 form), Bax, and Bcl-2. Quantitative analysis of (d) the ratio of cleaved-caspase-3 (p17 form) to pro-caspase-3 and (e) the ratio of Bax to Bcl-2 in heart tissues. Data are presented as mean ± SD, *n* = 12. ^∗^*P* < 0.05, ^∗∗^*P* < 0.01, and ^∗∗∗^*P* < 0.001. CLP = cecal ligation and puncture; rhMG53 = recombinant human mitsugumin-53.

It is generally accepted that abundant inflammatory mediators and excessive oxidative stress are crucial in caspase-3 activation and cardiomyocyte apoptosis, which contributes to SIMD [6]. As shown in Figures 7(a) and 7(b), the percentage of myocardial TUNEL-positive cells (stained brown) significantly increased 18 h after CLP (*P* < 0.01*vs.* sham group), which was significantly reduced by MG53 (*P* < 0.01*vs.* CLP group) and augmented by GW6471 (*P* < 0.05*vs.* CLP+rhMG53 group). As shown in Figures 7(c)–7(e), the expression of apoptosis-related proteins in the myocardium, the cleaved-caspase-3 to pro-caspase-3 and Bax to Bcl-2 ratio (both *P* < 0.01*vs.* sham group), sharply increased in SIMD rats and was prevented by MG53 (both *P* < 0.05*vs.* CLP group) and reversed by GW6471 (both *P* < 0.05*vs.* CLP+rhMG53 group).

## 4. Discussion

Sepsis is the leading cause of death among inpatients and is responsible for rising mortality and hospitalization rates worldwide every year [32]. It often leads to life-threatening multiple organ dysfunction, and the heart is one of the most commonly affected organs ^[^1^]^. SIMD is recognized as a major contributor to the prognosis of septic shock [33]. Previous studies [18–22, 34, 35] have demonstrated that MG53 is beneficial for treating cardiac diseases; however, studies primarily focusing on the effect of MG53 in SIMD are still relatively rare. In the present study, we established a SIMD model of rats and found a downregulation of endogenous MG53 and PPAR*α*, which was increased by exogenous rhMG53 in the myocardium 18 h after CLP. We further demonstrated that the supplementation of MG53 reduced myocardial oxidative stress and inflammatory injury and attenuated cardiomyocyte damage and apoptosis, which eventually improved cardiac function and survival rate in SIMD rats. To further clarify the protective mechanism of MG53, GW6471 (a PPAR*α* antagonist) was preprocessed. Our results suggest that the upregulation of PPAR*α* protein levels may be a possible mechanism for MG53 conferred myocardial protection in SIMD.

MG53 (also known as TRIM72) is a skeletal and cardiac muscle-specific TRIM-family protein that facilitates plasma membrane repair [12]. We previously reported that MG53 alleviated HIRI-induced oxidative stress and hepatocyte apoptosis by being anchored by dysferlin [19]. MG53 mediates cardiac cell membrane damage induced by MI [18], ischemia-reperfusion injury, [19, 20], or aortic valve disease [22]. It interacts with p85 and CaV3 and activates the prosurvival RISK pathway both in cardiac IPC and IPO [20, 21]. MG53 recently exhibited an anti-inflammatory function in cardiac hypertrophy by inhibiting the nuclear transfer of p65 and *β*-catenin, thereby downregulating their downstream target genes, including ANP, BNP, and *β*-MHC [22, 35]. Insufficient expression of MG53 plays a critical role in a range of myocardial pathological processes [18–22, 34, 35], and systemic delivery of exogenous recombinant MG53 protein ameliorates cardiomyocyte injury [18, 21, 35]. However, there have been few studies regarding MG53 on SIMD. Herein, we established a SIMD model and confirmed that the endogenous MG53 levels were insufficient after CLP. rhMG53 promoted the expression of MG53. Our results were similar to those of our previous study regarding HIRI [19] and those of Liu et al. regarding MI [18]. Moreover, we found the PPAR*α* protein levels were downregulated after CLP and elevated by MG53. It was in keeping with studies regarding DCM that MG53 overexpression led to the transcriptional upregulation of PPAR*α* [23]. Our findings suggest that MG53 may have an effect by upregulating PPAR*α* in SIMD.

SIMD is defined as the intrinsic, reversible, global impairment in cardiac function induced by sepsis [5]. Decreased systolic and diastolic myocardial contractility are the clinical features of SIMD [33]. Troponins and CK-MB levels are often elevated in SIMD patients or laboratory animals [36]. In the present study, the CLP group exhibited an impaired systolic (LVSP and +dp/dt_max_) and diastolic (LVEDP and -dp/dt_max_) heart function accompanied by histological change and cTnI, CK-MB, and LDH elevation in serum. MG53 improved sepsis-induced cardiac function impairment and cardiomyocyte damage, which was abolished by GW6471. In this study, the myocardial H-FABP levels decreased after CLP but were increased by MG53 supplement and suppressed by GW6471. FABPs are a class of small cytoplasmic proteins that facilitate the transfer of fatty acids (FAs) between extra- and intracellular membranes. H-FABP is abundant in myocardial cells [37] and is released from cytoplasm at almost the onset of myocardial injury [30]. Chen and Li [38] reported that circulating H-FABP levels may serve as biomarkers of organ dysfunction in patients with sepsis. Fan et al. [39] identified the upregulation of serum H-FABP in a mouse model of SIMD. Nevertheless, we chose to detect H-FABP levels in the myocardium rather than serum. This was partly because H-FABP levels in the serum can be disturbed when skeletal muscles are damaged or renal clearance is impaired [40]. The most important, H-FABP selectively cooperates with PPAR*α* and enables it to exert biological functions [41]. Our results further indicated that PPAR*α* may participate in the process of MG53 improving SIMD.

PPAR*α* is a nuclear receptor that regulates the expression of genes involved in FAs metabolism, lipoprotein synthesis, and metabolism [42]. When bound with endogenous or exogenic lipid/lipid metabolite ligands, PPAR*α* is activated, which heterodimerizes with retinoid X receptor (RXR) and binds to PPAR response elements (PPRE); the specific region of the DNA sequence is located in the promoters of target genes. Then, the transcription by recruiting RNA polymerase II (RNA Pol II) or other transcription factors was initiated; thus, PPAR*α* regulates gene function [43]. Under normal conditions, fatty acid oxidation (FAO) provides almost 70% of adenosine triphosphate (ATP) for energy production in cardiomyocytes [6]. Sepsis inhibits intracellular FAO, reduces cardiomyocyte ATP production, and eventually injures myocardial function [44]. It has been reported that *Ppara^−/−^* mice cannot sustain the elevated heart function and energy supply necessary to compensate for sepsis pathophysiology. PPAR*α* expression supports the hyperdynamic cardiac response and increased FAO in SIMD [24]. However, a sequence of studies has suggested that PPAR*α* exerts a protective effect in myocardial injury, not just by improving lipid utilization [45].

In the current study, we found that MG53 alleviated myocardial oxidative stress and inflammation damage in SIMD. The myocardial collagen volume stained by Masson's trichrome significantly increased 18 h after CLP operation, and MG53 could reduce the myocardial collagen volume. Additionally, we discovered that the protective function of MG53 may be connected to PPAR*α* upregulation. GW6471 reversed the protective effects of MG53 in SIMD. Even preoperatively injected with rhMG53, the myocardial oxidative indicators, the DHE fluorescent intensity, and serum proinflammatory cytokine (IL-1*β*, IL-6, and TNF-*α*) levels significantly increased in the GW6471 preconditioning group. Our results were consistent with those of Lou et al. [46], who found that PPAR*α* exhibited antioxidant and anti-inflammatory effects by upregulating antioxidativeindicators and downregulating oxidative indicators and proinflammatory cytokine expression. PPAR*α* has also been implicated in potent antioxidant effects by enhancing endothelial nitric oxide synthase and the release of nitric oxide [47]. It exhibited an anti-inflammatory effect through the inhibition of proinflammatory signaling pathways, such as nuclear factor-*κ*B (NF-*κ*B) in vascular injury [48]. The PPAR*α* knockout mice could not maintain appropriate immune functions against sepsis shock [49]. Nonetheless, how PPAR*α* reduced SIMD-induced oxidative stress and inflammation damage deserves further study.

It has been proven that cardiomyocyte apoptosis and caspase-3 activation contribute to SIMD [6]. The overproduction of reactive oxygen species (ROS) and inflammatory mediators plays a critical role in this pathological process [6]. In the current study, we found that the MG53 supplement alleviated cardiomyocyte apoptosis, inhibited caspase-3 activation, and downregulated the ratio of Bax to Bcl-2 in SIMD. This finding was similar to those of our previous study regarding MG53 in HIRI [19]. Intravenous injection of rhMG53 inhibited the toll-like receptor 4 (TLR4)/NF-*κ*B pathway and attenuated neurocyte apoptosis in LPS-induced neurotoxicity [50]. Furthermore, we found that GW6471 reversed the effect induced by MG53, suggesting that PPAR*α* may participate in the antiapoptotic effect induced by MG53 in SIMD. The activation of the sirtuin 1 (Sirt1)/PPAR*α* pathway participated in trimetazidine-mitigated cardiomyocyte apoptosis in SIMD [51]. Therefore, the underlying antiapoptotic mechanism of MG53/PPAR*α* in SIMD requires further investigation.

It has been reported that the overexpression of MG53 decreases glucose uptake and facilitates free FAs uptake in DCM and that this deleterious effect is connected to increased PPAR*α* expression [23]. As a nuclear receptor regulating lipid metabolism, the leading cause of the detrimental effect exerted by PPAR*α* is that excess lipid accumulation surpassed FAO leading to the overproduction of ROS and lipotoxicity in cardiomyocytes [23, 45]. We suspected that both MG53 and PPAR*α* played conflicting roles due to the different patterns of energy supply in SIMD and DCM. Therefore, energetic starvation in cardiomyocytes is a critical mechanism responsible for SIMD. Although we found MG53 protecting SIMD by upregulating PPAR*α*, the explicit mechanism is ambiguous. The nutrient utilization (glucose and FAs) and the underlying pathway between MG53 and PPAR*α* in SIMD require further investigation.

**Figure 8:**
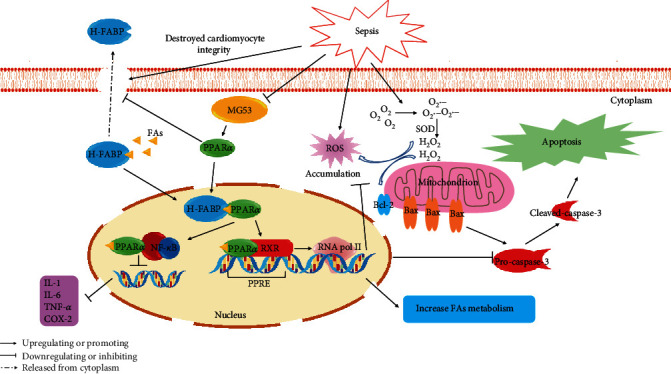
Schematic of the proposed mechanism involved in the protective effects of MG53 on PPAR*α* in SIMD rats. Sepsis caused the H-FABP released from cardiomyocyte plasma and downregulated MG53 and PPAR*α* protein levels. Endogenously, ROS were overproduced resulting in the activation of the caspase-3-dependent apoptosis pathway. Also, large amounts of inflammatory cytokines (IL-1, IL-6, and TNF-*α*) were generated due to the activation of some proinflammatory pathways (such as NF-*κ*B) after SIMD. The supplementation of MG53 upregulated PPAR*α*, attenuated cardiomyocyte damage, and prevented H-FABP release. H-FABP facilitated FAs transfer and activated PPAR*α*. PPAR*α* exhibited an anti-inflammatory effect through the inhibition of proinflammatory signaling pathways, such as NF-*κ*B. Also, PPAR*α* heterodimerized with RXR, banded to PPRE, and then recruited RNA Pol II to regulate gene function to play a crucial part in antioxidant stress, antiapoptosis, and promoting FAs metabolism. RXR = retinoid X receptor; RNA Pol II = RNA polymerase II; PPRE = PPAR response element; FAs = fatty acids.

In conclusion, we discovered a previously unreported relationship between MG53 and PPAR*α* in SIMD (Figure 8). The supplementation of MG53 upregulated PPAR*α*, reduced myocardial oxidative stress and inflammatory injury, attenuated cardiomyocyte damage and apoptosis, and eventually improved cardiac function and survival rate in SIMD rats. GW6471 preprocessing abolished the protective effects of MG53. Additionally, this study provides a new treatment strategy for SIMD.

## Data Availability

The datasets generated during and/or analyzed during the current study are available from the corresponding author on reasonable request.
